# Improved Glucose Control and Reduced Body Weight in Rodents with Dual Mechanism of Action Peptide Hybrids

**DOI:** 10.1371/journal.pone.0078154

**Published:** 2013-10-22

**Authors:** James L. Trevaskis, Christine M. Mack, Chengzao Sun, Christopher J. Soares, Lawrence J. D’Souza, Odile E. Levy, Diane Y. Lewis, Carolyn M. Jodka, Krystyna Tatarkiewicz, Bronislava Gedulin, Swati Gupta, Carrie Wittmer, Michael Hanley, Bruce Forood, David G. Parkes, Soumitra S. Ghosh

**Affiliations:** Amylin Pharmaceuticals LLC, San Diego, California, United States of America; University of Ulster, United Kingdom

## Abstract

Combination therapy is being increasingly used as a treatment paradigm for metabolic diseases such as diabetes and obesity. In the peptide therapeutics realm, recent work has highlighted the therapeutic potential of chimeric peptides that act on two distinct receptors, thereby harnessing parallel complementary mechanisms to induce additive or synergistic benefit compared to monotherapy. Here, we extend this hypothesis by linking a known anti-diabetic peptide with an anti-obesity peptide into a novel peptide hybrid, which we termed a phybrid. We report on the synthesis and biological activity of two such phybrids (AC164204 and AC164209), comprised of a glucagon-like peptide-1 receptor (GLP1-R) agonist, and exenatide analog, AC3082, covalently linked to a second generation amylin analog, davalintide. Both molecules acted as full agonists at their cognate receptors *in vitro*, albeit with reduced potency at the calcitonin receptor indicating slightly perturbed amylin agonism. In obese diabetic *Lep^ob^/Lep* ^*ob*^ mice sustained infusion of AC164204 and AC164209 reduced glucose and glycated haemoglobin (Hb_A1c_) equivalently but induced greater weight loss relative to exenatide administration alone. Weight loss was similar to that induced by combined administration of exenatide and davalintide. In diet-induced obese rats, both phybrids dose-dependently reduced food intake and body weight to a greater extent than exenatide or davalintide alone, and equal to co-infusion of exenatide and davalintide. Phybrid-mediated and exenatide + davalintide-mediated weight loss was associated with reduced adiposity and preservation of lean mass. These data are the first to provide *in vivo* proof-of-concept for multi-pathway targeting in metabolic disease via a peptide hybrid, demonstrating that this approach is as effective as co-administration of individual peptides.

## Introduction

Type 2 diabetes is a complex polygenic disorder currently affecting the lives of over 170 million people worldwide, with that number estimated to double by 2030 [1]. It is characterized by insulin resistance, impaired glucose-stimulated insulin release (pancreatic β-cell dysfunction), and inappropriate secretion of glucagon, which manifested in concert results in chronic hyperglycemia. Type 2 diabetes is strongly associated with the presence of obesity, and when observed in the presence of other disorders such as cardiovascular disease, hypertension and/or dyslipidemia, is known as Metabolic Syndrome [2]. Generally, the presence of three out of five of the following clinical signs would signify presence of Metabolic Syndrome: fasting blood glucose ≥100 mg/dL, elevated blood pressure with systolic ≥130 mm Hg and/or diastolic ≥85 mm Hg, triglycerides ≥150 mg/dL, reduced high-density lipoprotein cholesterol <40 mg/dL (males) or <50 mg/dL (females), and obesity (defined as country-specific waist circumference or body mass index) [2]. Established treatments for type 2 diabetes (insulin therapy, sulfonylureas, thiazolidinediones, metformin, or combinations of these) have not been completely successful at lowering hemoglobin A1c levels (Hb_A1c_) sufficiently or counteracting against the continued decline of pancreatic function in the diabetic state [3,4]. Furthermore, such therapies have been ineffective at combating the concomitant presence of other metabolic disorders, and are often associated with weight gain and other undesirable consequences [5]. Future pharmacotherapies for diabetes should therefore target not only glycemic control, but also the regulation of other factors such as weight loss, lipemic control, and/or preservation of pancreatic function. 

Recently, a new class of diabetes drug targeting the incretin system has emerged as an alternate form of diabetes therapy. Glucagon-like peptide-1 (GLP-1) and glucose-dependent insulinotropic polypeptide (GIP) are endogenous incretin hormones produced by the gut that elicit Hb_A1c_- and glucose-lowering effects via glucose-dependent insulinotropism, slowing of gastric emptying and suppression of post-prandial glucagon secretion [6]. Treatment with GLP-1 receptor (GLP-1R) agonists such as exenatide (exendin-4) or liraglutide significantly improves diabetic status with the added benefit of body weight reduction [7-9]. Exenatide treatment may also exert beneficial effects on pancreatic function [10], and has mediated improvements in hypertension [11] and cardiovascular function [12,13]. Compounds that prolong GLP-1 activity via inhibition of the processing enzyme dipeptidyl peptidase-4 have also been shown to exert moderate effects to lower glucose, but without significant beneficial effects on body weight [14]. 

Amylin is co-secreted with insulin from pancreatic β-cells in response to nutrient ingestion, and works co-operatively to regulate post-prandial hyperglycemia [15]. Whereas insulin stimulates peripheral glucose uptake, amylin’s glucose-lowering effects are primarily mediated via its actions of inhibiting gastric emptying, suppressing glucagon release, and inhibiting food intake [15]. The human amylin analogue pramlintide is approved for use as an antihyperglycemic agent (US; Food and Drug Administration) in patients with type 1 or 2 diabetes treated with mealtime insulin [16]. Clinical trial data have shown that, similar to exenatide treatment, pramlintide therapy is associated with modest weight loss [17]. Davalintide is a 32 amino acid amylinomimetic peptide which shares 49% identity to rat amylin and pramlintide. It displays significantly enhanced potency and efficacy for producing weight loss in rodent models compared to rat amylin [18], along with superior pharmaceutical properties when compared to pramlintide. Davalintide displays similar *in vitro* binding affinity to rat amylin in a rat amylin receptor preparation (nucleus accumbens), 10-fold greater affinity compared to rat amylin at the human calcitonin gene-related peptide-1 (CGRP) receptor and 100-fold greater affinity than rat amylin at the rat calcitonin (CT) receptor. Davalintide and amylin showed comparable potency to activate cAMP production in RINm5F cells [18].

With the goal of not only controlling diabetes but targeting improvements in Metabolic Syndrome, we leveraged the incretin and amylin classes of therapeutic hormones to create a molecule that could generate equal or better glucose control compared to exenatide with the added benefit of enhanced weight loss. Here we demonstrate for the first time the conjugation via natural or unnatural amino acid linkers of two peptide hormone receptor agonists, namely a GLP-1R agonist (AC3082, corresponding to exenatide_1-28_) and a second generation amylin analog, davalintide, into single chemical entities. We have termed these chemical entities as phybrids (*p*eptide *hybrids*). Two AC3082:davalintide phybrids, AC164204 and AC164209, that contain a Gly-Gly-Gly and a β-Ala-β-Ala linking moiety respectively, exhibited dual receptor agonism *in vitro* and induced distinctive *in vivo* properties of each parent peptide. AC164204 and AC164209 improved glucose tolerance and reduced Hb_A1c_ levels in diabetic rodents, coupled with body weight loss greater than that achievable with single administration of the parent peptide. AC164204 and AC164209 are therefore single molecular entities that can harness the anti-diabetic properties of incretin monotherapy with significantly greater weight loss in a single compound, thus potentially meeting the greatly unmet medical need for pharmacological interventions for Metabolic Syndrome.

## Materials and Methods

### Ethics statement

All studies were approved by the Institutional Animal Care and Use Committee at Amylin Pharmaceuticals, Inc., in accordance with Animal Welfare Act guidelines. 

### Chemistry materials

Lys-SCH_2_CH_2_CO-Leu-OCH_2_-Pam resin with a loading of 0.55 mmol/g was obtained from Rapp Polymere (Tübingen, Germany). Rink amide resin with a loading of 0.69 mmol/g was obtained from NovaBiochem (Gibbstown, NJ). Prefilled amino acid cartridges for the ABI 433A synthesizer were purchased from Midwest Biotech (Fishers, IN). Fmoc and Boc protected amino acids were obtained from EMD Biosciences (Billerica, MA). Side chain protection groups for Fmoc-amino acids were Fmoc-Arg(Pbf), Fmoc-Trp(Boc), Fmoc-Glu(OtBu), Fmoc-Lys(Boc), Fmoc-Gln(Trt), Fmoc-Tyr(tBu), Fmoc-Ser(tBu), Fmoc-Thr(tBu), Fmoc-His(Trt), Fmoc-Cys(Trt), Fmoc-Cys(Acm), Fmoc-Asn(Trt) and Fmoc-Asp(OtBu). Side chain protecting groups for Boc-amino acids were Arg(Tos), Asp(OcHex), Asn(Xan), Glu(OcHex), His(DNP), Lys(2-ClZ), Ser(Bzl), Thr(Bzl), Trp(For), Tyr(BrZ). 2-(1H-benzotriazol-1-yl)-1,1,3,3-tetramethyluronium hexafluorophosphate (HBTU), and were obtained from Peptide International (Louisville, KY). Pseudoprolines were purchased from Genzyme Pharmaceuticals. Trifluoroacetic acid (TFA) was obtained from Halocarbon Products (River Edge, NJ). Piperidine was obtained from Spectrum Chemicals (*Gardena*, CA). 2-mercaptoethanol (ME) was obtained from MP Biomedicals (Solon, OH). N,N-diisopropylethylamine (DIEA), m-cresol, p-cresol, triisopropylsilane (TIS), thiophenol and tris(2-carboxylethyl)phosphine hydrochloride (TCEP) were purchased from Acros Organics (Geel, Belgium). N,N-dimethylformamide (DMF), N-methylpyrrolidone (NMP), dichloromethane (DCM) and HPLC grade acetonitrile were obtained from Fisher Scientific (Pittsburgh, PA). HF cleavage was performed by CS Bio Company (Menlo Park, CA).

### Peptide synthesis, purification and analysis

Peptides were synthesized on ABI 433A synthesizers with either Boc or Fmoc chemistry. Boc chemistry employs Boc protecting group at the N-terminus of each amino acid. The peptides were synthesized by attaching the first C-terminal Boc protected amino acid to the solid support, which was then followed by cycles of Boc removal by TFA and coupling to the carboxylic acid of the subsequent Boc-amino acid residue for the elaboration of the full peptide sequence. Fmoc chemistry employs Fmoc (fluorenylmethyloxycarbonyl) protecting group at the N-terminus of each amino acids, which is removed by a base (usually 20% piperidine in DMF) before the next coupling cycle. Preparative reverse-phase HPLC was performed on a Waters HPLC/MS system consisting of a Waters 2525 Prep HPLC Pump, 2767 sample manager, 2487 dual absorbance detector and Micromass ZQ mass spectrometer. Analytical reverse-phase HPLC was performed on an Agilent 1100 system equipped with a 6120 quadrupole LC/MS. Phybrids were prepared initially by native chemical ligation method and later also with linear synthesis using Fmoc chemistry.

### Native chemical ligation method

Segment I thioesters were prepared on a 433A peptide synthesizer employing t-Butylcarbonyl (*t*-Boc) chemistry with *in situ* neutralization protocol as described [19]. Standard Boc amino acids and a Lys-SCH_2_CO-Leu-OCH_2_-Pam resin [20] with a 0.55 mmol/g loading were used. The peptide thioesters were cleaved using standard HF cleavage condition with *p*-cresol as scavenger. Formyl (on Trp25) and DNP (on His1) groups remained on the peptide and were removed after the ligation reaction. The crude peptides were purified on Waters preparative HPLC/MS instrument with a Kromasil 250 x 21.2 mm C4 column using a linear gradient (25-45%) of buffer B in buffer A over 30 min (buffer A = 0.05% TFA in water; buffer B = 0.05% TFA in ACN) and a flow rate of 20 mL/min. The fractions were analyzed by the Agilent analytical HPLC/MS and the pure fractions were pooled and lyophilized. A typical 0.2 mmol scale synthesis yielded 90.0 mg of segment I peptide. Segment II peptide was synthesized using standard FastMoc chemistry on Rink amide resin with 0.69 mmol/g loading. The peptide cleavage was carried out using TFA/*m*-cresol/TIS/H_2_O (15/2/1/2, v/v). Following *t*-butyldimethylether precipitation and lyophilization, the peptide was purified in a similar manner as segment I. A typical 0.25 mmol scale synthesis yielded 114.5 mg of segment II peptide.

Segment I and II peptides (molar ratio *ca*. 1.2:1) were dissolved in 6 M Guanidine-HCl, 300 mM sodium phosphate, pH 7.5 at concentrations up to 5 mg/ml. Thiophenol was added (1% v/v) to catalyze the ligation reaction. The reaction was stirred under nitrogen at room temperature and its progress was monitored by Agilent analytical HPLC/MS. The reaction was complete after 4 hrs. β-mercaptoethanol was added (20% v/v) to the reaction mixture to remove the DNP group and to dissolve the thiophenol dimer precipitate. The pH was adjusted to 8-8.5 using 10N NaOH. Piperidine was added (20% v/v) to remove the formyl protecting group. The reaction mixture was diluted four-fold with low pH buffer (6 M GndHCl, 300 mM NaOAc, 10 mM TCEP-HCl, pH 4.0) before loading the mixture onto the C4 column. The final ligated product was purified in a manner similar to that of segments I and II. Pure fractions were selected by analytical HPLC/MS, pooled and lyophilized. The ligated peptide was dissolved in 20% acetic acid (5-7 mg/ml) and oxidized with an iodine solution (0.125 M in AcOH). A saturated thiosulfate solution was added to quench unreacted iodine. The oxidized cyclic peptide was then purified as described above to give pure phybrid. A typical 20.0 μmol scale ligation yielded 42.0 mg of phybrid. 

### Linear Fmoc synthesis method

Phybrids were also synthesized using standard FastMoc chemistry with optimizations on Fmoc-Pal-PEG-amide resin. Optimizations include, a) double couplings at positions R20, L21, F22, and I23; b) pseudoproline dipeptides were used at residues L10 and S11. Cleavage and purification steps were similar to that described above. A typical 0.2 mmol scale synthesis yielded 11 mg of phybrid.

***Invitro* receptor function assays**. Receptor function was measured using the cAMP dynamic 2 kit (CisBio, Bedford, MA) according to the manufacturer’s instructions. Peptides and HEK-293 C1a6 cells (overexpressing the human calcitonin receptor) or 6-23 rat thyroid carcinoma cells (endogenously expressing the GLP-1 receptor) were combined and incubated for 30 min at room temperature. The detection reaction was incubated at room temperature for 2 h. cAMP content was measured using a TECAN fluorometer plate reader (San Jose, CA). Values represent mean from multiple independent experiments.

### Experimental animals and study design

Animals were housed individually in standard caging at 22°C in a 12-hour light, 12-hour dark cycle. 

### Acute single-dose studies

For glucose-lowering analysis, non-fasted male, conscious NIH Swiss mice (33-36 g; n=9-10/group) received compound (30 nmol/kg, i.p.) at t=0 and blood was collected at t=20 min. For assessment of effects on plasma calcium levels, fed male anesthetized Sprague Dawley rats (340-370 g, n=3-4/group) received compound (2 nmol/kg, s.c.) at t=0 and blood was collected at t=120 min. For rat intravenous glucose tolerance tests fed male anesthetized Sprague Dawley rats (n=4-6 per group) were catheterized via the femoral artery to monitor arterial pressure, for saline infusion for fluid maintenance, and sample collection; via the femoral vein for infusion of saline, test compound, and glucose bolus. At t=-30 min, 1 hour after completion of surgical procedure, saline or test compound (30 pmol/kg/min) intravenous infusion was started and continued throughout the experiment. At t=0, an intravenous bolus of 5.7 mmol/kg of D-glucose was administered over 1-1.5 min. The data for exenatide was adapted from [21] and is used with permission.

### Acute dose-response studies

For oral glucose challenge, peptides were injected i.p. into 4 h-fasted female NIH Swiss mice (n=9-16/group) 5 min prior to glucose gavage (1.5 g/kg). Blood was collected at t=30 min and glucose measured. 

### Chronic studies

For dose-response studies examining the effect of phybrids on glucose and glycated hemoglobin (Hb_A1c_) in mice, male leptin-deficient *Lep^ob^/Lep* ^*ob*^ mice (Jackson Laboratories, Bar Harbor, ME; n=10 per group) were randomized into treatment groups matched for baseline Hb_A1c_ levels, and subcutaneously implanted with two Alzet osmotic mini-pumps (Durect Corporation, Cupertino, CA) infusing either vehicle (50% DMSO, 0.1% bovine serum albumin), AC164204 (30 or 300 nmol/kg/d), AC164209 (30 or 300 nmol/kg/d), davalintide (30 or 300 nmol/kg/d), exenatide (2.4 nmol/kg/d), or exenatide + davalintide (2.4 and 300 nmol/kg/day, respectively). Mice were treated for 28 days. Blood samples were collected at day -1 and at termination for analysis of blood glucose and Hb_A1c_ levels. For dose-response studies examining the effect of phybrids in diet-induced obese (DIO) rats, male DIO-prone rats (Charles River Laboratories, Wilmington, MA) were maintained on a moderately high fat diet (32% kcal fat; Research Diets D1226B, New Brunswick, NJ) for 10 weeks before and during treatment. DIO-prone rats (body weight 489 ± 3 g; n=6 per group) were subcutaneously implanted with two Alzet osmotic mini-pumps (Durect Corporation) containing either vehicle (50% DMSO), AC164204 or AC164209 (3, 10, 30 and 100 nmol/kg/d), exenatide (7.2 nmol/kg/d), davalintide (2.8 nmol/kg/d), or exenatide + davalintide (7.2 and 2.8 nmol/kg/d, respectively). Rats were treated for 28 days. Blood was collected after an overnight fast on day 14. On day 28 a terminal blood sample was taken by cardiac puncture and the animals were euthanized.

### Body composition

Fat and lean mass of conscious DIO rats was determined (Echo Medical Systems, Houston, TX) at baseline and termination. Adiposity (fat mass/body weight x 100) and percent lean tissue (lean mass/body weight x 100) was calculated.

### Plasma analyses

Blood glucose was measured using a glucometer (OneTouch Ultra, LifeScan, Milpitas, CA). Plasma triglyceride, total cholesterol, HDL cholesterol, calcium and Hb_A1c_ were measured using an Olympus AU400e Bioanalyzer (Olympus America, Irving, TX). Plasma insulin was measured by ELISA (Crystal Chem, Downer’s Grove, IL), or radioimmunoassay (Rat Insulin RIA Kit #RI-13K, Millipore Corporation, St. Louis, MO). AC164204 and AC164209 concentrations were assessed by validated sandwich ELISA developed at Amylin Pharmaceuticals, LLC. 

### Data analysis

Significant peptide treatment effects (p<0.05) were identified by one-way ANOVA with Neumann-Keuls post hoc test. Analyses were performed and graphs generated using Prism 4 (Graphpad Software, San Diego, CA). All data points are expressed as mean ± SEM.

## Results

### Design and Syntheses of AC164204 and AC164209

**Figure 1:**
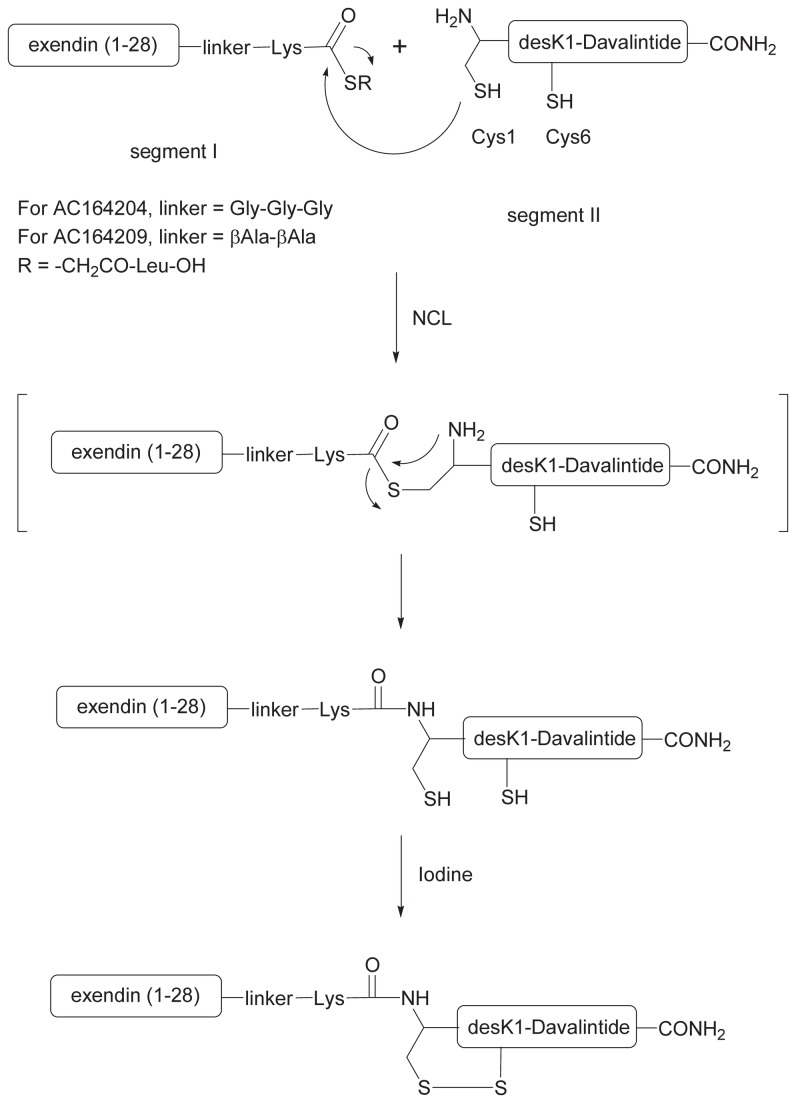
General reaction scheme for the preparation of AC164204 and AC164209. Two peptide segments I and II were used. Segment I is a thioester that contains AC3082 sequence, a linker portion and a Lysine at the C-terminus. Segment II is uncyclized desK1-Davalintide with a free cysteine at the N-terminus to provide a reactive moiety for the native chemical ligation. The linker in segment I is Gly-Gly-Gly for AC164204 and β-Ala-β-Ala for AC164209. The sequence of AC3082/exendin(1-28) is HGEGTFTSDLSKQMEEEAVRLFIEWLKN and the sequence of desK1-davalintide is CNTATCVLGRLSQELHRLQTYPRTNTGSNTY.

AC164204 and AC164209 are phybrids composed of the GLP-1R agonist AC3082 and the amylinomimetic peptide davalintide [18], that are linked by a β-Ala-β-Ala or Gly-Gly-Gly spacer respectively. Natural and unnatural amino acid linkers were incorporated to assess if peptidase cleavage at these sites would have an impact on metabolic activity. AC3082, a truncated exendin-4 analog, is comparable to exendin-4 in its *in vitro* and *in vivo* activities, and was selected as a phybrid building block because its shorter length facilitates ease of synthesis of the target molecules. Our previous work on the two peptide receptor agonist families had indicated that it is important to preserve the C-terminal amide functionality of an amylinomimetic and to avoid N-terminal modifications of the GLP-1R agonist for optimal retention of biological activity (unpublished results). Based on these findings, the GLP-1R agonist was positioned at the N-terminus of davalintide. The two phybrids were prepared by the using native chemical ligation (NCL) approach [22,23]. The NCL strategy for AC164204 and AC164209 is illustrated in Figure 1, wherein each molecule is assembled from two peptide segments. Segment I is a C-terminal thioester whose sequence comprises AC3082, a linker and a terminal lysine residue at the C-terminus. Segment II is uncyclized des-(Lys)^1^-Davalintide, whose sequence starts with a free cysteine on the N-terminus. Our synthetic design took advantage of this N-terminal cysteine to serve as a handle for the native chemical ligation. In line with previous reports [22,23], the other free cysteine in des-(Lys)^1^-Davalintide does not interfere in the ligation reaction. Once the two segments were ligated, an oxidation reaction was used to form the disulfide bridge and yield the final phybrid. 

### *In vitro* receptor activity assays

**Figure 2:**
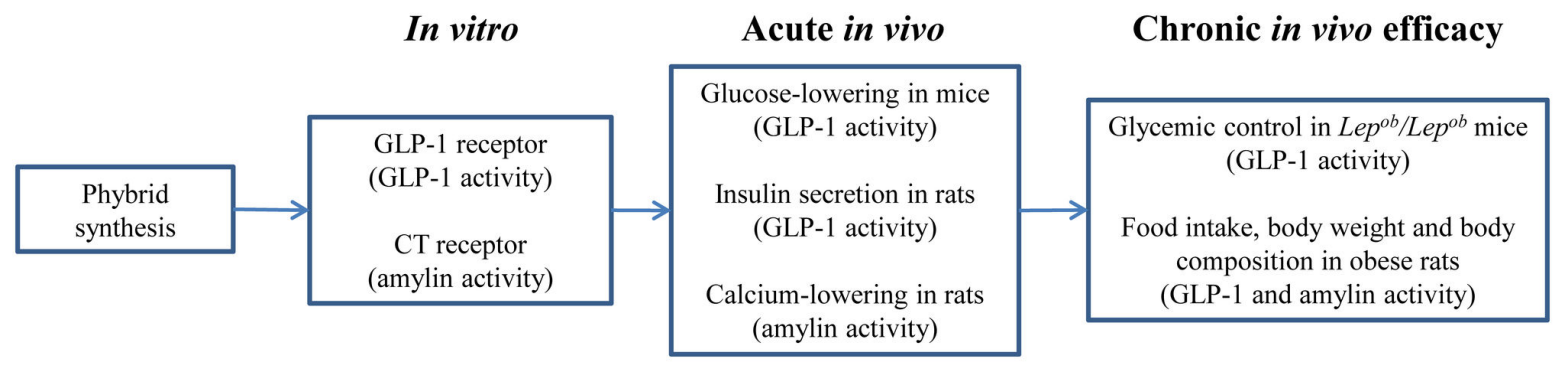
Flowchart of phybrid characterization of receptor activity and *in* *vivo* efficacy. After synthesis, the ability of the GLP-1 and amylinomimetic portions of the phybrid to activate their appropriate receptors was assessed: activation of cAMP in cells expressing the GLP-1R or the CT receptor. Acute *in* *vivo* characteristics of GLP-1 and amylin physiology were then assessed: glucose-lowering in mice for GLP-1 activity, insulin secretion in rats for GLP-1 activity and calcium-lowering in rats for amylin activity. In chronic efficacy models to more accurately gauge the ability of the phybrids to render equal or superior metabolic control relative to mono- or dual-therapy we utilized two models: obese, diabetic mice to assess impact on chronic glucose control, and DIO rats to assess impact on food intake, body weight and body composition.

After phybrid synthesis, we embarked on a thorough characterization process beginning with confirmation of receptor activity *in vitro*, signature acute activity assays *in vivo* and ultimately with chronic efficacy studies in rodent models (Figure 2). First we tested the ability of phybrid compounds to activate the GLP-1 and the calcitonin (CT) receptors. The CT receptor serves a surrogate receptor for ascertaining amylinomimetic functional activity [18]. AC164204 and AC164209 activated both GLP-1 and CT receptors, albeit at a slightly reduced potency compared to its parent peptides (Table 1). Of note, phybrids comprised of the same parent compounds but covalently attached with alternate linkers such as (β-Ala)_4_, (Gly)_6_ and (Pro)_6_, demonstrated an ability to fully agonize the GLP-1 and CT receptors (Table 1). However, the *in vitro* activities and acute plasma glucose lowering effects (Table 2) were comparable to that of AC164204 and AC164209, suggesting that additional length or rigidity of the linker does not lead to an improvement in ligand-mediated receptor activation. These data confirm that AC3082:davalintide phybrids can activate both functional receptors *in vitro*.

**Table 1:** *In vitro* measurement of cAMP responses to administration of peptides.

**Peptide**	**Linker**	**GLP-1 receptor (EC_50_, nM)**	**Amylin/calcitonin receptor (EC_50_, nM)**
Exenatide	NA	0.004	>1000
AC3082	NA	0.01	>1000
Davalintide	NA	>1000	0.05
AC164204	Gly-Gly-Gly	0.05	1.27
AC164209	β-Ala-β-Ala	0.10	3.20
AC164831	β-Ala-β-Ala-β-Ala-β-Ala	0.03	1.65
AC164835	Gly-Gly-Gly-Gly-Gly-Gly	0.14	1.20
AC164836	Pro-Pro-Pro-Pro-Pro-Pro	0.07	1.61

NA, not applicable; EC_50_, half-maximal effective concentration.

**Table 2:** Plasma glucose and calcium changes following acute peptide injection in mice (glucose) and rats (calcium).

**Peptide**	**Glucose (%)^a^**	**Calcium (%)^b^**
AC3082	-40.5 ± 2.1*	-0.3 ± 0.3
Davalintide	18.5 ± 5.3*	-16.2 ± 1.3*
AC164204	-43.0 ± 1.3*	-15.4 ± 1.3*
AC164209	-40.7 ± 3.1*	-14.2 ± 1.0*
AC165835	-32.4 ± 1.4*	-19.8 ± 2.3*
AC165836	-44.4 ± 2.3*	-18.6 ± 6.0*

^a^,% of control at t=20 min; ^b^,% of baseline at t=2 h; *p<0.05 vs. control.

NA, not applicable; EC_50_, half-maximal effective concentration.

### *In vivo* assessment of functional biology

**Figure 3:**
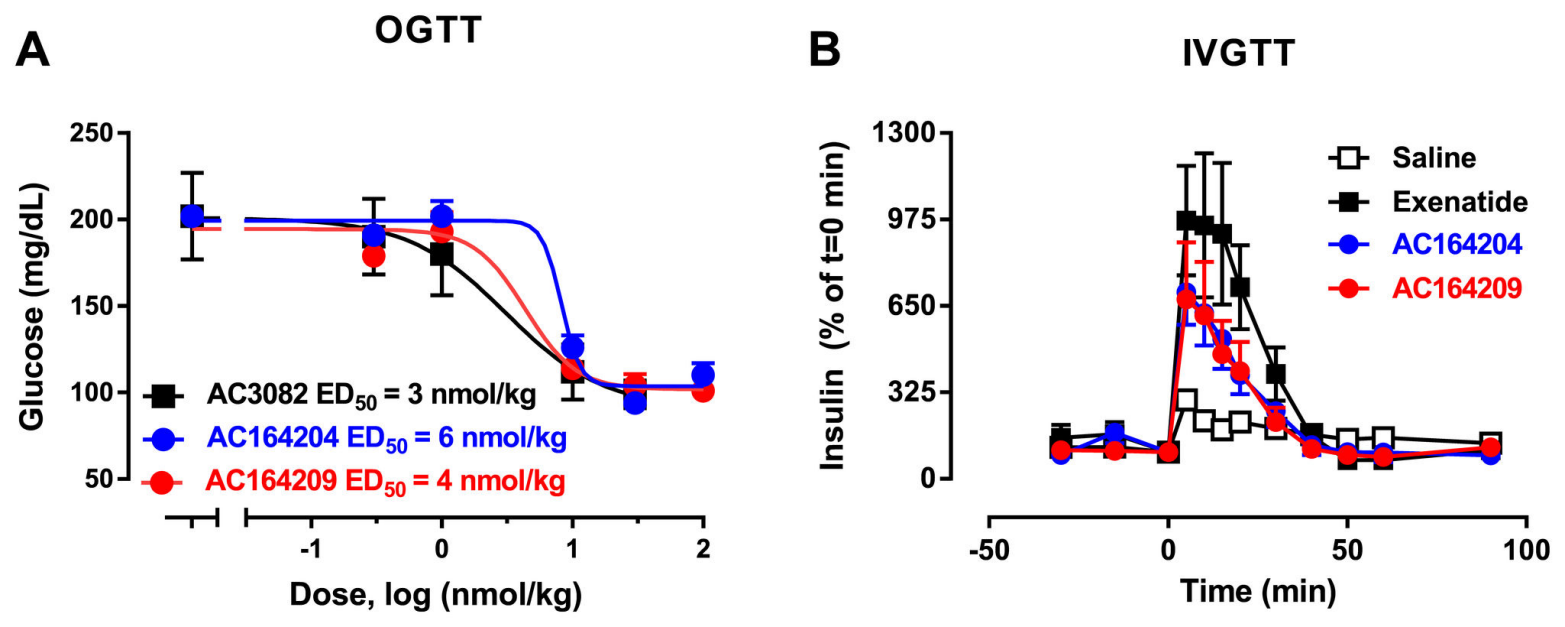
Phybrid compounds reduced 30 min plasma glucose concentrations similar to GLP-1 receptor agonist (AC3082) in non-diabetic mice, and were insulinotropic in Sprague Dawley rats. (**A**) Oral glucose tolerance test (OGTT) of AC3082, AC164204 and AC164209 comparing potency and efficacy of both compounds; (**B**) intravenous glucose tolerance test (IVGTT) showing glucose-dependent insulinotropism of exenatide, AC164204, and AC164209.

To confirm dual pharmacology of both AC164204 and AC164209 *in vivo*, we examined acute glucose-lowering (to confirm AC3082-like activity) in non-fasted mice and calcium-lowering (to confirm davalintide-like activity) in non-fasted rats. AC164204 and AC164209, similar to AC3082 but not davalintide administration, significantly reduced 20-min blood glucose compared to vehicle (Table 2). Furthermore, both phybrids were able to dose-dependently lower glucose levels 30 min after an oral glucose challenge in normal mice (ED_50_ = 4-6 nmol/kg), with similar potency and efficacy as AC3082 (ED_50_ = 3 nmol/kg; Figure 3A). The glucose-stimulated insulinotropic properties were also assessed in rats relative to exenatide. Both AC164204 and AC164209 demonstrated increased glucose-stimulated insulin release at 30 pmol/kg/min, however at slightly reduced ability relative to exenatide (Figure 3B). Plasma calcium, a marker of amylin agonism, was reduced similarly by AC164204, AC164209 and davalintide whereas AC3082 administration did not affect plasma calcium levels 2 h post-injection (Table 2). 

### Sustained infusion of AC164204 or AC164209 reduces Hb_A1c_, food intake and body weight in diabetic mice

**Figure 4:**
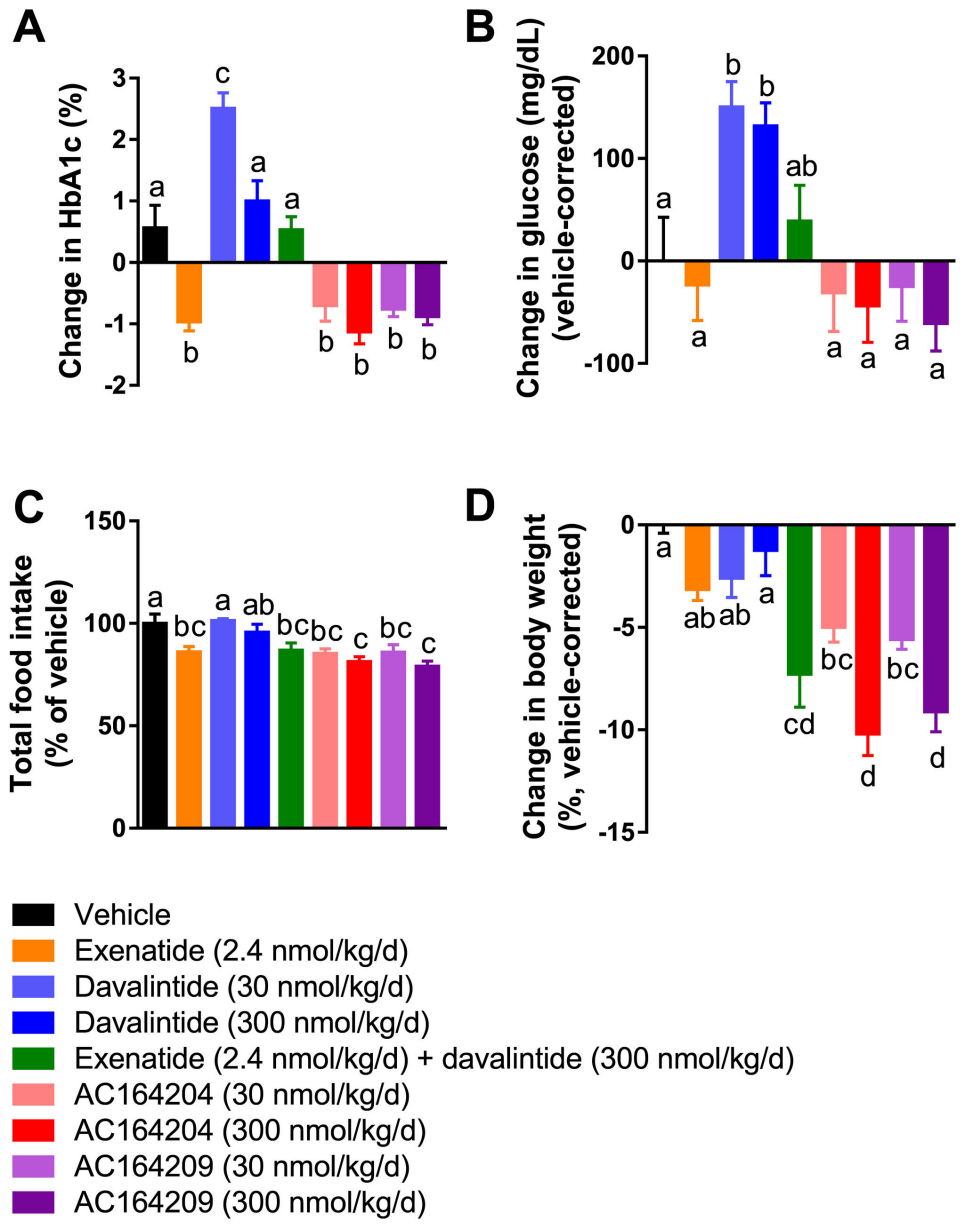
Phybrid peptides induced greater weight loss compared to single peptide treatment and improved glycemic control similar to GLP-1 receptor agonism in obese diabetic *Lep^ob^/Lep* ^*ob*^ mice. (**A**) Change in HbA1c, (**B**) change in plasma glucose, (**C**) total food intake, expressed as a percentage of vehicle, and (**D**) change in body weight following subcutaneous peptide infusion in leptin-deficient *Lep^ob^/Lep* ^*ob*^ mice for 28 days. Groups not sharing the same superscript letter were significantly different from each other (p<0.05).

We examined the effects of 28 days of sustained infusion of AC164204 and AC164209 on Hb_A1c_ and glucose concentrations, as well as food intake and body weight, in diabetic *Lep^ob^/Lep* ^*ob*^ mice. Exenatide infusion significantly reduced HbA1c relative to vehicle- and davalintide-treated *Lep^ob^/Lep* ^*ob*^ mice (Figure 4A, B). In rodent models, but not in human subjects, amylin analogs are known to elicit a paradoxical species-specific increase in glucose levels [15]. Davalintide increased Hb_A1c_ at the lower dose only, and increased glucose concentrations at both low and high doses, compared to vehicle; co-administration of exenatide and davalintide induced an intermediate effect such that both Hb_A1c_ and glucose concentrations were not significantly different from vehicle (Figure 4A, B). Importantly, AC164204 and AC164209, similar to exenatide, decreased Hb_A1c_ values relative to vehicle- and davalintide-treated mice (Figure 4A, B). 

Total cumulative food intake was inhibited by administration of exenatide and exenatide + davalintide, but not davalintide alone (Figure 4C). Both AC164204 and AC164209 at the upper dose reduced food intake compared to vehicle or davalintide-treated *Lep^ob^/Lep* ^*ob*^ mice (Figure 4C). Inhibition of food intake corresponded with reductions in body weight (Figure 4D). At these doses neither exenatide nor davalintide significantly reduced body weight compared to vehicle, however exenatide + davalintide significantly reduced body weight compared to exenatide or davalintide alone. High dose AC164204 and AC164209 significantly reduced body weight compared to vehicle, exenatide- and davalintide-treated *Lep^ob^/Lep* ^*ob*^ mice, but equivalently to exenatide + davalintide. 

### Sustained infusion of AC164204 or AC164209 reduces food intake and body weight in DIO rats

**Figure 5:**
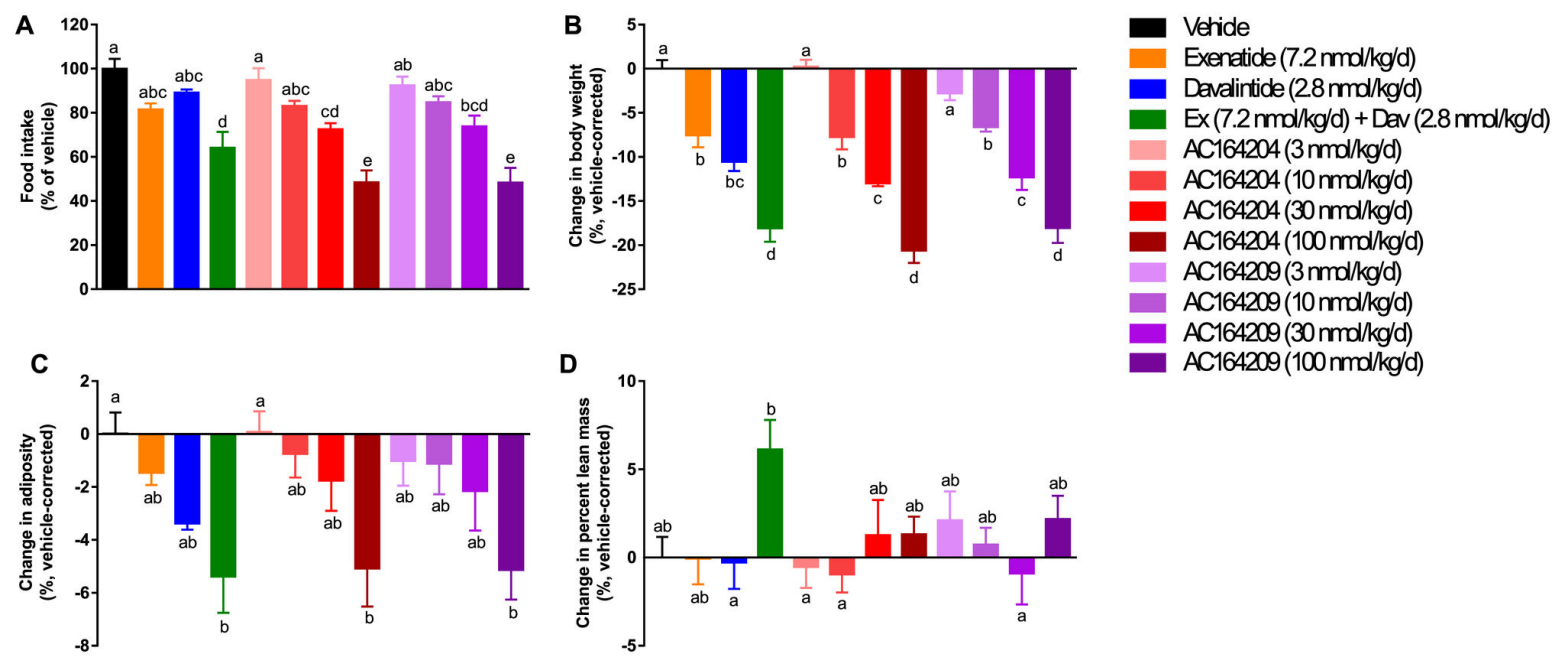
Sustained infusion of AC164204 or AC164209 to diet-induced obese rats dose-dependently inhibited food intake, and reduced body weight and fat mass. (**A**) Total cumulative food intake, (**B**) percent change in body weight, (**C**) change in adiposity, and (**D**) change in percent lean mass after 28 days of peptide infusion. Groups not sharing the same superscript letter were significantly different from each other (p<0.05).

To assess the ability of anti-diabetic phybrids to reduce body weight and inhibit food intake in a second preclinical model, we examined the effect of sustained subcutaneous infusion of both phybrids, parent compounds alone or co-infused parent compounds in non-diabetic DIO rats. For this experiment we used exenatide as the GLP-1R agonist. Dose-dependent and durable reductions in total food consumption over the 28-day treatment period were observed with both AC164204 and AC164209 treatment (Figure 5A). Combination treatment with exenatide and davalintide, but not single peptide administration, also reduced food intake. The highest doses of AC164204 and AC164209 significantly reduced food intake beyond that induced by co-administration of parent peptides (Figure 5A). Both AC164204 and AC164209 dose-dependently reduced body weight, with a maximum vehicle-corrected weight loss of ~20%. The highest dose of each phybrid significantly reduced body weight compared to single administration of each parent compound (Figure 5B). Exenatide plus davalintide also significantly reduced body weight compared to each parent compound and matched the weight loss induced by the highest dose of phybrid (Figure 5B). AC164204 or AC164209 (100 nmol/kg/day) and exenatide plus davalintide induced greater loss of fat mass relative to vehicle (Figure 5C). While loss of body weight was associated with a small degree of lean mass reduction (data not shown), the percentage of lean mass to body weight was not significantly affected (Figure 5D). 

Terminal non-fasting plasma analysis from DIO rats revealed no between-group differences in glucose, total cholesterol or HDL cholesterol, with a trend for reduced triglyceride concentrations as phybrid dose increased (Table S1). Dose-dependent increases in AC164204 and AC164209 plasma concentrations were evident after 14 days of treatment; after 28 days higher variability in plasma exposure of phybrids was evident at the two highest doses (Table S1). 

## Discussion

To the best of our knowledge, our findings are the first to detail the construction and development of a phybrid with dual or multiple pro-metabolic functions *in vivo*, and to demonstrate durable anti-diabetic and anti-obesity effects of these molecules in rodent models. Our phybrids were designed from unrelated peptide hormones, and hence are distinct from peptide chimeras that are derived from parent molecules with significant sequence similarities. Our work on davalintide, a chimera of amylin and salmon-calcitonin, is illustrative of the chimeric concept [18]. The data presented here strongly support the hypothesis that the phybrids AC164204 and AC164209 are novel single chemical entities able to mediate the key pharmacological properties of its parent hormones. Phybrid effects were equal or superior to co-infusion of parent peptides, and superior to either parent peptide alone. The β-Ala-β-Ala linker in AC164209 was incorporated to prevent peptidase cleavage at the linker site. This concern was unfounded as AC164204, which contains a Gly-Gly-Gly spacer, is comparable to AC164209 in its metabolic stability (data not shown) and in its *in vivo* activity profile. 

 The most prevalent disorder associated with type 2 diabetes is overweight and/or obesity. The emerging incretin class of anti-diabetes agents such as exenatide have demonstrated promising long-term therapeutic potential, not merely by combating hyperglycemia, but by also exerting significant weight loss in clinical trials [8,24,25]. Combination therapy has been proposed as a way to generate greater weight loss than achieved by the current class of anti-diabetic treatments. The recently approved weight loss drug Qsymia, a fixed dose combination of phentermine and topiramate induced close to 10% body weight loss over 12 months in obese subjects, highlights the clinical potential of combination therapies (reviewed in 26). Furthermore, co-administration of amylin or pramlintide with the adipocyte hormone leptin induced significantly greater weight loss (12-13%) than either amylin/pramlintide or leptin alone in obese rats and humans [27,28]. Combinatorial approaches are likely to be the best way to approach the degree of weight loss seen with surgical interventions, however combination approaches for diabetes that elicit similar weight loss are lacking. GLP-1-based combinations able to exert meaningful weight loss have only, to date, been reported in preclinical models. Several versions of dual GLP-1R and glucagon receptor dual agonist chimeras have been reported to induce significant weight loss in rodents [29-32]. These molecules have been elegantly designed as single peptide dual agonists with varying degrees of potency at each of the parent receptors and chemically modified to increase stability and half-life, but no human data are currently available. Of note, GLP-1 and glucagon are derived from the same preproglucagon gene [33], and are thus a uniquely positioned family that lends itself naturally to development as a chimera. 

We aimed to create a superior anti-obesity and anti-diabetic agent by harnessing two separate, but validated, pharmacological agents in a single molecular entity. In DIO rats, amylin has been reported to act in concert with molecules such as phentermine and sibutramine [34], peptide YY (PYY) [35], melanocortin 4 receptor agonist [36], and cholecystokinin [37,38], leading to enhanced weight loss. Combining amylin with these agents all resulted in at least additive effects on body weight and food intake. Furthermore, combined administration of amylin with the adipocyte-derived hormone leptin induced a synergistic interaction for both body weight loss and food intake in obese rats [28,39], which was recapitulated in two human clinical trials [27,28]. Exenatide has been successfully utilized in combination with metformin, sulfonylurea or thiazolidinedione in human clinical populations [7,8,40]. Acute interactions of exenatide and amylin on food intake in non-human primates were recently reported by Bello et. al. [41]. In this well-designed multi-dose combination study using response surface methodology, the investigators determined that exenatide and amylin induced typically greater than additive effects on food intake [41]. Data presented here are, to our knowledge, the first demonstration of additive metabolic benefit with combined GLP-1R and amylin agonism in a chronic setting in rodents, and combined with the reported primate data, suggest that the mechanisms underlying this interaction may be preserved across species.

One limitation of any phybrid is that these covalently linked linear structures have, by necessity, a fixed 1:1 stoichiometry. Therefore, preservation of potency at the receptor is critical. Exenatide is a highly potent GLP1-R agonist, as is the AC3082 peptide utilized here as part of AC164204 and AC164209. Likewise, davalintide is a highly potent molecule at the amylin/calcitonin receptor, a proxy *in vitro* marker of amylin activity [18]. In phybrid form, both AC164204 and AC164209 were full agonists at each receptor, albeit with somewhat reduced potency. Reduced potency may explain why the phybrids exhibited reduced insulinotropism; however, phybrids were equally efficacious as AC3082 for glucose-lowering/oral glucose tolerance acutely, and efficacy at the amylin-dependent action (calcium-lowering) was unaffected relative to davalintide. The long-term durability of AC164204 and AC164209 was examined by sustained infusion studies in rodent models of metabolic disease. In these studies both phybrids exerted approximately equal glucoregulatory properties as exenatide alone, and induced significantly more weight loss than single administration of maximally efficacious doses of exenatide or davalintide. These effects were, however, observed only at significantly higher doses of compound than required for exenatide or davalintide treatment suggesting that while loss of potency with a phybrid molecule may not hinder acute responses it may be more of a consideration when implementing long-term treatment strategies. 

 The mechanisms underlying the interaction between GLP-1R and amylin agonism in a pharmacological setting remain to be elucidated. Both hormones trigger central and peripheral physiological responses that contribute to their anorexigenic and weight-reducing effects, however each is mediated by discrete and independent receptor/neuronal pathway activation [42]. Whether combination treatment enhances these existing processes or prevents counter-regulatory responses to enable greater efficacy is unknown. Nevertheless, both amylin and GLP-1 pathways have a proven record of success when leveraged in a combinatorial approach for the alleviation of metabolic disorders. With dual GLP-1R and amylin agonists we have harnessed the integrated and neurohormonal control of diabetes, and potentially obesity and related disorders, in a single molecular entity.

## Supporting Information

Table S1 **Plasma glucose, lipid and phybrid concentrations following sustained administration of AC164204, AC164209, exenatide, and/or davalintide in DIO rats.** Glucose, triglycerides, total cholesterol and HDL cholesterol were measured after 28 days of sustained infusion. Groups not sharing a superscript are significantly different from one another (p<0.05); ND, not determined.
(DOCX)
