# Supplementary material for: Improved Glucose Control and Reduced Body Weight in Rodents with Dual Mechanism of Action Peptide Hybrids
Source: PLoS One. 2013 Oct 22;8(10):e78154. doi: 10.1371/journal.pone.0078154 (PMC3805588; doi:10.1371/journal.pone.0078154)
Supplement: Table S1 — Plasma glucose, lipid and phybrid concentrations following sustained administration of AC164204, AC164209, exenatide, and/or davalintide in DIO rats. Glucose, triglycerides, total cholesterol and HDL cholesterol were measured after 28 days of sustained infusion. Groups not sharing a superscript are significantly different from one another (p<0.05); ND, not determined. (DOCX) [file pone.0078154.s001.docx]

| **Treatment** | **Glucose**  **(mg/dL)** | **Triglycerides**  **(mg/dL)** | **Cholesterol**  **(mg/dL)** | **HDL cholesterol**  **(mg/dL)** | **Phybrid (ng/mL)**  **14 days** | **Phybrid (ng/mL)**  **28 days** |
| --- | --- | --- | --- | --- | --- | --- |
| Vehicle | 135 ± 3 | 278 ± 38 | 131 ± 7 | 33 ± 1 | <0.2*^a^* | <0.2*^a^* |
| AC164204 (3 nmol/kg/d) | 126 ± 9 | 276 ± 56 | 125 ± 8 | 31 ± 2 | 4.2 ± 0.8*^a^* | 27.8 ± 20.5*^a^* |
| AC164204 (10 nmol/kg/d) | 130 ± 7 | 207 ± 19 | 127 ± 10 | 33 ± 1 | 15.2 ± 2.4*^a^* | 92.1 ± 70.5*^a^* |
| AC164204 (30 nmol/kg/d) | 125 ± 11 | 148 ± 13 | 116 ± 7 | 31 ± 1 | 54.2 ± 11.3*^a,b^* | 219.4 ± 80.7*^a^* |
| AC164204 (100 nmol/kg/d) | 119 ± 9 | 182 ± 24 | 132 ± 8 | 37 ± 2 | 191.0 ± 50.4*^c^* | 185.0 ± 65.4*^a^* |
| AC164209 (3 nmol/kg/d) | 133 ± 10 | 221 ± 28 | 131 ± 14 | 32 ± 1 | 4.9 ± 0.6*^a^* | 3.5 ± 0.9*^a^* |
| AC164209 (10 nmol/kg/d) | 116 ± 7 | 220 ± 32 | 120 ± 7 | 33 ± 3 | 13.0 ± 1.5*^a^* | 20.1 ± 10.0*^a^* |
| AC164209 (30 nmol/kg/d) | 120 ± 2 | 149 ± 14 | 115 ± 1 | 34 ± 1 | 69.1 ± 29.1*^b^* | 382.2 ± 275.2*^a^* |
| AC164209 (100 nmol/kg/d) | 118 ± 5 | 146 ± 25 | 112 ± 4 | 33 ± 2 | 209.2 ± 17.7*^c^* | 391.0 ± 179.2*^a^* |
| Exenatide (7.2 nmol/kg/d) | 145 ± 7 | 179 ± 11 | 112 ± 4 | 33 ± 1 | ND | ND |
| Davalintide (2.8 nmol/kg/d) | 124 ± 7 | 168 ± 26 | 131 ± 8 | 35 ± 1 | ND | ND |
| Exenatide (7.2 nmol/kg/d) + davalintide (2.8 nmol/kg/d) | 129 ± 10 | 186 ± 33 | 125 ± 6 | 37 ± 1 | ND | ND |

**Supplemental Table 1.** Plasma glucose, lipid and phybrid concentrations following sustained administration of AC164204, AC164209, exenatide, and/or davalintide in DIO rats. Glucose, triglycerides, total cholesterol and HDL cholesterol were measured after 28 days of sustained infusion. Groups not sharing a superscript are significantly different from one another (p<0.05); ND, not determined.
